# An Improved Toolkit of Gateway- and Gibson Assembly-Compatible Vectors for Protoplast Transfection and Agrobacterium-Mediated Plant Transformation

**DOI:** 10.21203/rs.3.rs-3558280/v1

**Published:** 2023-11-07

**Authors:** Jeffrey Allen, Michael Flanagan, Sunita Pathak, Samantha Emenecker, Ryan Emenecker, Lucia Strader

**Affiliations:** Duke University; Duke University; Duke University; Washington University in St. Louis; Washington University in St. Louis; Duke University

**Keywords:** Protoplast transfection, Agrobacterium transformation, transgenic plant, plant transformation, transient expression, live cell imaging, cloning, Gateway, Gibson-Assembly, fluorescence microscopy

## Abstract

**Objective:**

Understanding the regulation and function of plant genes is essential for addressing the challenges faced by modern agriculture. Plant transformation, in conjunction with fluorescence microscopy, offers a powerful approach to investigate the dynamic behavior of plant genes and the proteins they encode. We previously developed a set of Gateway-compatible tissue-specific plant transformation vectors. In this paper we aim to expand the toolkit of vectors available for Agrobacterium-mediated plant transformation and protoplast transfection.

**Results:**

Here, we introduce new Agrobacterium-mediated plant transformation vectors by introducing additional fluorophores to create the pJRA vector series. Additionally, we introduce the pLCS series of vectors, a new set of modular Gateway- and Gibson assembly-compatible vectors designed for protoplast transfection. All described vectors are available from Addgene to serve as a resource for the plant research community.

## Background

Understanding how genes regulate plant biology is crucial to addressing the challenges faced by modern agriculture. Plant transformation is a powerful tool to investigate how plant genes regulate biology. When combined with fluorescence microscopy, transgenic plants allow for live imaging of proteins in their native cellular environment. Previously, we developed a series of Gateway-compatible tissue-specific plant transformation vectors [1]. Here we expand the toolkit of available Agrobacterium-mediated plant transformation vectors with additional fluorophores and extend the set to include a set of modular Gateway- and Gibson assembly-compatible vectors for protoplast transformation.

### pLCS Protoplast Vectors

**Figure 1:**
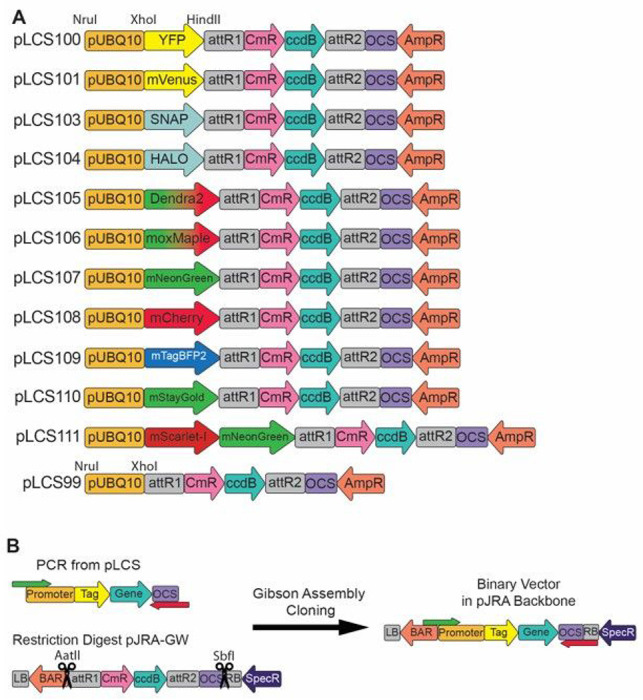
pLCS Series Protoplast Vectors The pLCS vector series is compatible with both Gateway cloning and Gibson assembly. **A** pLCS100-111 feature the *UBQ10* promoter (pUBQ10) driving the expression of a fluorophore in frame with a gateway cassette consisting of *attR*1 and *attR*2 recombination sites, the chloramphenicol resistance gene (CmR), and the ccdB gene. The gateway cassette is followed by the terminator sequence of the octopine synthase gene (OCS) and the ampicillin resistance gene (AmpR) as a selectable marker. pLCS99 has no fluorescent tag to allow for the creation of custom vectors. The *UBQ10* promoter is flanked by *Nru*I and *Xho*I restriction sites. The fluorescent protein tags are flanked by *Xho*I and *Hind*III restriction sites. **B** pLCS protoplast vectors can be converted to binary pJRA vectors. PCR primers JA_298_UBQ_HiFi-F and JA_299_GW_HiFi-R (Table S1) are used to linearize the expression cassette with an added 20-bp of homology to pJRA-GW at each end. The pJRA-GW backbone is linearized by restriction digest using *Aat*II and *Sbf*I. The linearized fragments are then used for NEB HiFi cloning or Gibson Cloning according to the manufacturers protocol. This cloning strategy was used to convert the various pLCS vectors into pJRA vectors.

Protoplasts are a powerful system for transient expression of plant genes. Protoplasts are well suited to synthetic biology, allowing for a rapid cycle of design, testing, and iteration [2]. We created a collection of protoplast expression vectors known as the pLCS vector series. Each pLCS vector carries the *UBIQUITIN10 (UBQ10)* promoter, which typically drives the expression of *UBQ10*, a uniform and widely-expressed transcript in Arabidopsis [3]. In plants, the *UBQ10* promoter displays a more uniform expression pattern than many commonly used viral promoters, such as the CaMV *35S* promoter. In each pLCS vector, the *UBQ10* promoter drives the expression of a fluorescent protein (FP), followed by a Gateway cassette (*attR1*, chloramphenicol resistance gene, *ccdB* gene, *attR2*), and the octopine synthase (OCS) terminator. The backbone of pLCS vectors contains the *AmpR* gene, which encodes beta-lactamase and confers resistance to ampicillin and carbenicillin in bacteria (Fig. 1A). Thus, a Gateway LR clonase reaction with an entry vector produces a vector that ultimately express the protein of interest with an N-terminal fluorescent protein tag.

We first created pLCS100 by subcloning the *UBQ10* promoter, a gene encoding *EYFP*, the gateway cassette, and OCS terminator from pUBQ10-YFP-GW [1] into the pUC19 [4] backbone. As EYFP is a weak dimer, and dimerization can create artifacts that influence the behavior of the tagged protein, we then created pLCS101 by replacing EYFP with monomeric mVenus [5] (Fig. 1).

mNeonGreen is an exceptionally bright monomeric fluorescent protein with an excitation and emission spectra between that of GFP- and YFP-derived fluorescent proteins [6]. To take advantage of this exceptionally bright fluorophore, we created pLCS107, which contains mNeonGreen in place of mVenus (Fig. 3A). StayGold is a recently developed highly photostable and bright FP enabling long imaging times with minimal photobleaching [7]. To take advantage of this new FP, we created pLCS110. StayGold is a dimer and users should be aware of potential artifacts due to its dimerization. During the preparation of this manuscript, a monomeric version of StayGold, known as mStayGold, containing the mutation E138D, was described in a preprint [8]. Whereas the data shown herein were collected using the dimeric StayGold, we have deposited the updated version of pLCS110 containing the monomeric mStayGold with Addgene.

To enable multi-color imaging experiments in co-transformed protoplasts, we created pLCS108 containing the FP mCherry [9]. Additionally, we created pLCS109 containing a gene encoding mTagBFP2, the brightest blue fluorophore currently available [10]. mTagBFP2 can be used to serve as a donor for Forster resonance energy transfer experiments (FRET) when paired with mNeonGreen, such as that carried by pLCS107.

To enable tracking of specific pools of protein and super resolution microscopy (e.g., PALM), we created pLCS105 and pLCS106 by inserting the genes encoding the green-to-red photoswitchable proteins DENDRA2 or moxMaple3, respectively [11, 12] (Fig. 3C). Similarly, pLCS103 and pLCS104 have a SNAP-tag or HaloTag respectively. SNAP-tag and HaloTag combine the advantage of being genetically encoded with the exceptional brightness of synthetic dyes, at the expense of labeling time [13]. Both the SNAP-tag and HaloTag, as well as various ligands, have been used successfully in plants (Fig. 3A) [14, 15].

pLCS111 carries a gene encoding a tandem fluorescent protein timer (tFT) consisting of mScarlet-I fused to mNeonGreen. mNeonGreen matures very rapidly (~ 10 mins), whereas mScarlet-I, a fast-maturing variant of mScarlet, matures more slowly (~ 26 mins) [16]. By fusing the mScarlet-I-mNeonGreen tFT to a protein of interest, it is possible to determine the age of the fusion protein based on the ratio of red-to-green fluorescence [17, 18] (Fig. 3E). It is important to note that the N-terminus of a protein is often a major determinant of protein stability due to the N-end rule [19–21]. As the default orientation for fusions in pLCS111 are N-terminal fusions it is prudent to test in the opposite orientation. Care should be taken when interpreting these data and appropriate controls should be used [22].

Finally, pLCS99 has no fluorescent protein to enable construction of custom protein fusions driven by the *UBQ10* promoter. The pLCS series was designed to be modular, thus the *UBQ10* promoter in each vector is flanked by a *NruI* and *XhoI* restriction site allowing the *UBQ10* promoter to be swapped easily using Gibson assembly or restriction ligation cloning. Similarly, the fluorescent protein in each vector is flanked by *XhoI* and *HindIII* restriction sites.

### pJRA Binary Vectors

**Figure 2:**
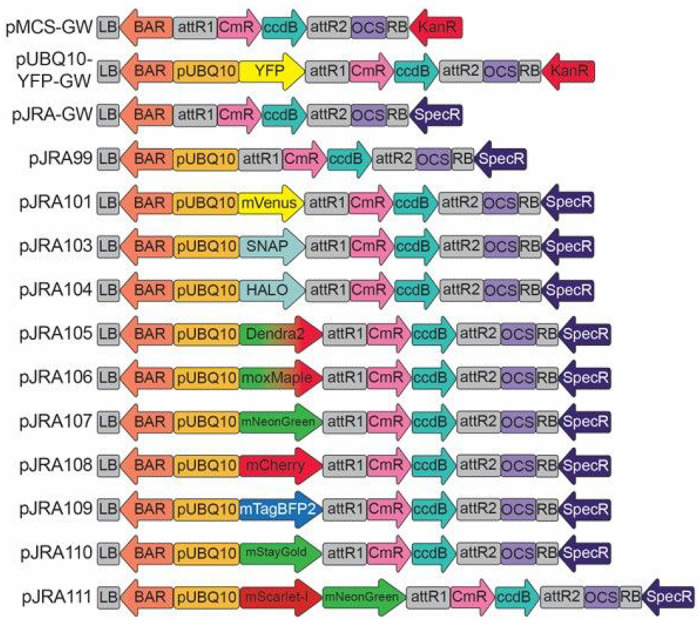
pJRA-GW was created from pMCS-GW [1] by replacing the kanamycin resistance gene (KanR) with the spectinomycin resistance gene (SpecR). The pJRA vectors are binary vectors designed for agrobacterium-mediated transformation and improve upon the previously published pMCS-GW and pUBQ10-YFP-GW vectors. Between the left and right borders (LB and RB) the BASTA resistance gene (BAR) is flanked by the manopine synthase promoter and manopine synthase terminator providing resistance to BASTA in transformed plants. As in the pLCS series, the pUBQ10 drives the expression of the fluorophore in frame with the gateway cassette.

The original pMCS-GW and pUBQ10-YFP-GW vectors [1] while immensely useful, both contain kanamycin resistance genes. As most Gateway entry vectors, such as pENTR/D-TOPO, are also kanamycin resistant, it was necessary to linearize such entry vectors before performing an LR reaction. To remedy this problem, we replaced the kanamycin resistance gene with a spectinomycin/streptomycin resistance gene to create pJRA-GW. Taking advantage of the protoplast vectors previously created, the *UBQ10* promoter, FP and gateway cassette from each pLCS vector was inserted into the pJRA-GW backbone to create the pJRA series of vectors with the same fluorophores as each pLCS vector (Fig. 2). The pJRA series of vectors are well-suited to transient expression in tobacco and for creation of stable transgenic lines. In the latter case, transformed plants can be screened based on resistance to glufosinate/phosphinothricin (commonly referred to as BASTA^™^).

**Figure 3:**
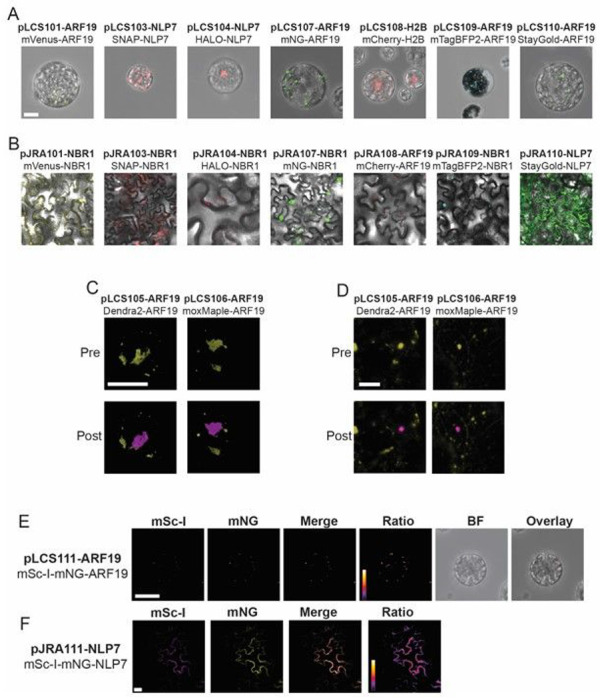
Transient Expression of pLCS Vectors in Protoplasts and pJRA Vectors in Tobacco Confocal microscopy of Arabidopsis protoplasts transformed with pLCS vectors and *Nicotiana benthamiana* leaves transformed using the pJRA vectors. **A-B** Images of various genes tagged with basic (constitutively fluorescent) tags in protoplasts (A) and tobacco (B). Individual channels are shown in Figure S1 & S2. **C** Close up images of 3D projected ARF19 condensates tagged with Dendra2 and moxMaple3 before (pre) and after (post) photoconversion by a 405 nm laser. D Images of NBR1 tagged with Dendra2 and moxMaple3. The yellow channel represents the green form and magenta the red form. **E** Ratiometric images of ARF19 tagged with the mScarlet-I-mNeonGreen (mSc-I-mNG) tandem fluorescent timer in protoplasts. As mNeonGreen has a faster maturation than mScarlet-I, the ratio mSc-I / mNG reflects the age of the protein (*i.e.,* a larger ratio equates to older protein). Scale bar 25 μm. F Ratiometric images of NLP7 tagged with the mScarlet-I-mNeonGreen tandem fluorescent timer in tobacco leaves.

Each vector in the pJRA series was used for transient expression in *Nicotiana benthamiana* to test their functionality (Fig. 3). Though fluorescence was visible for all constructs, high background was observed in the case of pJRA103 due to unbound SNAP-Cell TMR-Star, even after multiple washes. Curiously, this problem was not observed in Arabidopsis protoplasts transformed with pLCS103 (Fig. 3A), nor in tobacco transformed with pJRA104 when labeled with HALO-TMR (Fig. 3B). This discrepancy suggests that the SNAP-Cell TMR-Star ligand may have difficulty penetrating the cell wall or cuticle of intact plant cells. Further optimization of the treatment and washing procedure is likely needed to ensure the ligand enters the cells and all unbound dye is removed, particularly when dealing with intact plant tissues.

## Conclusion

In summary, we have expanded the toolkit of plant transformation vectors available to plant researchers interested in understanding the regulation and function of plant genes. The pLCS series of protoplast vectors and the pJRA series of binary vectors offer researchers additional tools for investigating plant gene and protein behavior using live cell imaging. Several kanamycin resistant “legacy” binary vectors and the tissue specific vectors we previously published [1] have also been deposited with Addgene (Table S5). These vectors, shared through Addgene, will serve as valuable tools for the plant research community and enable new discoveries in the field.

## Materials and Methods

### pLCS Vector Construction

pLCS100 was created by generating a restriction fragment from pUBQ10-YFP-GW containing the *UBQ10* promoter, YFP the gateway cassette and OCS terminator, then subsequently ligating that fragment into the pUC19 backbone [4]. To create pLCS101, mVenus was PCR amplified with *Xho*I and *Hind*III restriction sites at the 5’ and 3’ end respectively, then subcloned into pCR4 using Zero Blunt TOPO cloning according to the manufacturer’s recommendation. pLCS100 and pCR4-mVenus were then digested with *Xho*I and *Hind*III. mVenus was then ligated into the linearized pLCS backbone in-frame with Gateway recommendation sites using Quick Ligase (NEB). pLCS103-107 were created using the same strategy.

pLCS108-111 were created using NEB HiFi cloning. In all cases pLCS101 was linearized by PCR to exclude the existing mVenus sequence. The linearized backbone was then treated with *Dpn*I to digest any template plasmids. The mCherry sequence was amplified by PCR, whereas mTagBFP2, StayGold, and mScarlet-I-mNeonGreen were synthesized by Twist Bioscience. In all cases, the tags contained 20-bp overlaps with the pLCS backbone for HiFi cloning according to the manufacture’s recommendation. To create the untagged pLCS99, HiFi cloning was used to delete the mNeonGreen sequence from pLCS107. The entry clone containing the sequence of interest and the appropriate destination vector were used for LR Clonase reactions to create the final vector. Alternatively, the sequence of interest was PCR amplified with 15- to 20-bp of homology to the PCR linearized pLCS vector and used for NEB HiFi cloning. A full list of primers used for the creation and sequencing of these vectors can be found in Table S1-2.

### pJRA-GW Vector Construction

pMCS-GW was digested with *NsiI*and *PsiI* to excise the kanamycin resistance gene. A DNA fragment encoding the spectinomycin gene with 20-bp of homology to the digested pMCS-GW was synthesized by Twist Bioscience. The SpecR gene was then cloned into the pMCS-GW backbone using NEB HiFi cloning to create pJRA-GW. To convert pLCS vectors to pJRA vectors, pJRA-GW was linearized by restriction digest using *AatII* and *SbfI*. PCR was used to amplify the region containing pUBQ10, the FP Gateway cassette, and OCS terminator with 20 bp of homology at each end to the restriction digested pJRA backbone. The PCR fragment was then inserted into the pJRA backbone using HiFi cloning. The primer sequences used to create these vectors are in Table S1-2.

### Transient Expression in Tobacco

Electro-competent *Agrobacterium tumefaciens* strain GV3101 were transformed by electroporation [23]. Agrobacterium liquid cultures were grown overnight in LB media at 28 °C. Agrobacterium were then centrifuged at 1800xg for 3 minutes and resuspended in resuspension media (10 mM MES pH 5.6, 10 mM MgCl_2_, 150 μM acetosyringone) then grown for 2–4 hours at 28 °C. After incubation, the Agrobacterium solution was injected into *Nicotiana benthamiana* leaves using a syringe. Transformed *Nicotiana benthamiana* leaves were imaged 48 or 72 hours after infiltration [24].

### Transient Expression in Arabidopsis Protoplasts

Arabidopsis *Col-0* protoplasts were prepared using the tape sandwich method and transfected using PEG [25]. Protoplasts were then transfected using PEG mediated transformed. Transfected protoplasts were imaged the following day. A detailed procedure for protoplast isolation and transfection is imcluded in Additional File 6.

### SNAP and HALO-tag Labelling

Ligands, SNAP-Cell TMR-Star (NEB) or HALO TMR (Promega), were diluted in DMSO to 100 μM. For labelling in tobacco, small disks were cut from leaves infiltrated with Agrobacterium the prior day. The leaf disks were incubated in 1 μM ligand in 10 mM MES pH 5.6 with gentle shaking for one hour. The leaf disks were then washed overnight with 10 mM MES pH 5.6 to remove unbound dye. For labeling in Arabidopsis protoplasts, transformed protoplasts were incubated with 1 μM ligand in W5 solution for one hour. The protoplasts were centrifuged at 300 g for 3 mins and the supernatant was removed. The protoplasts were then washed twice with 1 ml W5 solution to remove unbound ligand.

### Microscopy

Protoplasts and tobacco leaves were imaged on a Leica SP8 confocal microscope. Images were processed using Fiji. For ratiometric calculations using the fluorescent timers (pLCS111 and pJRA111) the images were processed first by subtracting the background. The levels were adjusted to the same values in both channels. A Gaussian filter (radius = 1 for protoplasts, radius = 0.5 for tobacco) was applied, then the ratio between the channels was calculated by dividing the red (mSc-I) channel by the green (mNG) channel then multiplying by 127. For imaging constructs with photoconvertible tags (pLCS105-106 and pJRA105-106) the tagged proteins were photoconverted using a 405 nm laser.

### Limitations

Expression behind the strong, constitutive, UBQ10 promoter can result in greater protein levels than under normal physiological conditions. All the protein tags developed are at the N-terminus of fusion protein following an LR Clonase reaction. The position of protein tags can alter the behavior of the tagged protein; thus, it is prudent to also test the behavior of the expressed protein with the tag at the C-terminus.

## Data Availability

All described vectors are deposited with Addgene and are available for a small fee. All data are included in the article and its additional files.
